# Endoscopic mucosal resection (EMR) versus endoscopic submucosal dissection (ESD) for resection of large distal non-pedunculated colorectal adenomas (MATILDA-trial): rationale and design of a multicenter randomized clinical trial

**DOI:** 10.1186/s12876-016-0468-6

**Published:** 2016-05-26

**Authors:** Y. Backes, L. M. G. Moons, J. D. van Bergeijk, L. Berk, F. ter Borg, P. C. J. ter Borg, S. G. Elias, J. M. J. Geesing, J. N. Groen, M. Hadithi, J. C. H. Hardwick, M. Kerkhof, M. J. J. Mangen, J. W. A. Straathof, R. Schröder, M. P. Schwartz, B. W. M. Spanier, W. H. de Vos tot Nederveen Cappel, F. H. J. Wolfhagen, A. D. Koch

**Affiliations:** Department of Gastroenterology & Hepatology, University Medical Center Utrecht, Heidelberglaan 100, 3508 GA Utrecht, Netherlands; Department of Gastroenterology & Hepatology, Gelderse Vallei, Ede, Netherlands; Department of Gastroenterology & Hepatology, Sint Franciscus, Rotterdam, Netherlands; Department of Gastroenterology & Hepatology, Deventer Hospital, Deventer, Netherlands; Department of Gastroenterology & Hepatology, Ikazia, Rotterdam, Netherlands; Julius Center for Health Sciences and Primary Care, University Medical Center Utrecht, Utrecht, Netherlands; Department of Gastroenterology & Hepatology, Diakonessenhuis, Utrecht, Netherlands; Department of Gastroenterology & Hepatology, Sint Jansdal, Harderwijk, Netherlands; Department of Gastroenterology & Hepatology, Maasstad hospital, Rotterdam, Netherlands; Department of Gastroenterology & Hepatology, Leiden University Medical Center, Leiden, Netherlands; Department of Gastroenterology & Hepatology, Groene Hart Hospital, Gouda, Netherlands; Department of Gastroenterology & Hepatology, Máxima Medical Center, Eindhoven, Netherlands; Department of Gastroenterology & Hepatology, Gelre Hospital, Apeldoorn, Netherlands; Department of Gastroenterology & Hepatology, Meander Medical Center, Amersfoort, Netherlands; Department of Gastroenterology & Hepatology, Rijnstate hospital, Arnhem, Netherlands; Department of Gastroenterology & Hepatology, Isala, Zwolle, Netherlands; Department of Gastroenterology & Hepatology, Albert Schweitzer, Dordrecht, Netherlands; Department of Gastroenterology & Hepatology, Erasmus Medical Center, Rotterdam, Netherlands

**Keywords:** Colorectal adenoma, Endoscopic mucosal resection, Endoscopic submucosal dissection, Randomized clinical trial, Colonoscopy

## Abstract

**Background:**

Endoscopic mucosal resection (EMR) is currently the most used technique for resection of large distal colorectal polyps. However, in large lesions EMR can often only be performed in a piecemeal fashion resulting in relatively low radical (R0)-resection rates and high recurrence rates. Endoscopic submucosal dissection (ESD) is a newer procedure that is more difficult resulting in a longer procedural time, but is promising due to the high en-bloc resection rates and the very low recurrence rates. We aim to evaluate the (cost-)effectiveness of ESD against EMR on both short (i.e. 6 months) and long-term (i.e. 36 months). We hypothesize that in the short-run ESD is more time consuming resulting in higher healthcare costs, but is (cost-) effective on the long-term due to lower patients burden, a higher number of R0-resections and lower recurrence rates with less need for repeated procedures.

**Methods:**

This is a multicenter randomized clinical trial in patients with a non-pedunculated polyp larger than 20 mm in the rectum, sigmoid, or descending colon suspected to be an adenoma by means of endoscopic assessment. Primary endpoint is recurrence rate at follow-up colonoscopy at 6 months. Secondary endpoints are R0-resection rate, perceived burden and quality of life, healthcare resources utilization and costs, surgical referral rate, complication rate and recurrence rate at 36 months. Quality-adjusted-life-year (QALY) will be estimated taking an area under the curve approach and using EQ-5D-indexes. Healthcare costs will be calculated by multiplying used healthcare services with unit prices. The cost-effectiveness of ESD against EMR will be expressed as incremental cost-effectiveness ratios (ICER) showing additional costs per recurrence free patient and as ICER showing additional costs per QALY.

**Discussion:**

If this trial confirms ESD to be favorable on the long-term, the burden of extra colonoscopies and repeated procedures can be prevented for future patients.

**Trial registration:**

NCT02657044 (Clinicaltrials.gov), registered January 8, 2016.

## Background

Resection of colorectal adenomas has shown to lower the mortality rate due to colorectal cancer with 60 % [1]. Especially large adenomas maintain a high risk of progression to invasive cancer, underlining the importance of adequate resection [2]. Endoscopic resection of large adenomas has been proven to be feasible and safe, with less morbidity, mortality and costs compared to surgical resection [3]. Endoscopic resection of non-pedunculated adenomas is most often performed using the ‘lift-and-cut’ endoscopic mucosal resection (EMR) technique [4]. However, in adenomas larger than 2 cm in size EMR can often only be performed in a piecemeal fashion (pEMR) due to the limited size of the snare, difficulty to position the endoscope, and often extension of the polyp over one or multiple folds [5, 6]. Although safe, piecemeal resection lowers the reliability of assessing the dysplasia free resection margins (R0 resection) at histology. This is also reflected by the relative high recurrence rate at follow-up colonoscopy after EMR ranging between 12-16 %, and even increasing up to 30 % in non-pedunculated polyps exceeding 40 mm in size [7, 8].

For this reason endoscopic submucosal dissection (ESD) was developed in Japan, a technique that enables to achieve en-bloc resection even in large polyps [9]. Several retrospective observational studies compared EMR and ESD. A recent meta-analysis pooled the results yielding a total of 2299 lesions [10]. Although studies were biased by baseline differences with special regard to tumor location and polyp size due to lack of a randomized design, results are promising. Rates of en bloc resection and radical resection were much higher, and the rate of recurrence was much lower in the ESD-group (91.7, 80.3 and 0.9 % respectively) than in the EMR-group (46.7, 42.3 and 12.2 % respectively). This benefit comes at the expense of an about three-fold longer procedure time and more complications (perforation rate 5.7 % versus 1.4 %). However, this safety profile may be favorable in the distal colon, given the lower risk for complications as compared to proximally located polyps [11]. We hypothesize that the prolonged procedure time will initially result in higher healthcare costs, but that this time-investment will earn itself back on the long-term due a higher number of R0-resections and lower recurrence rates with less need for repeated procedures. If indeed ESD proves to be (cost-)effective on the long-term, burden of extra colonoscopies and repeated procedures can be prevented for patients in the future. It is important to address this gap in the current knowledge, especially now that screening programs have been widely introduced and the detection of large adenomas will further increase.

Therefore, the aim of this randomized clinical trial is to evaluate the effectiveness and cost-effectiveness of ESD against EMR on both short (i.e. 6 months) and long-term (i.e. 36 months) for large (>20 mm) distal non-pedunculated adenomas.

## Methods

**Fig. 1:**
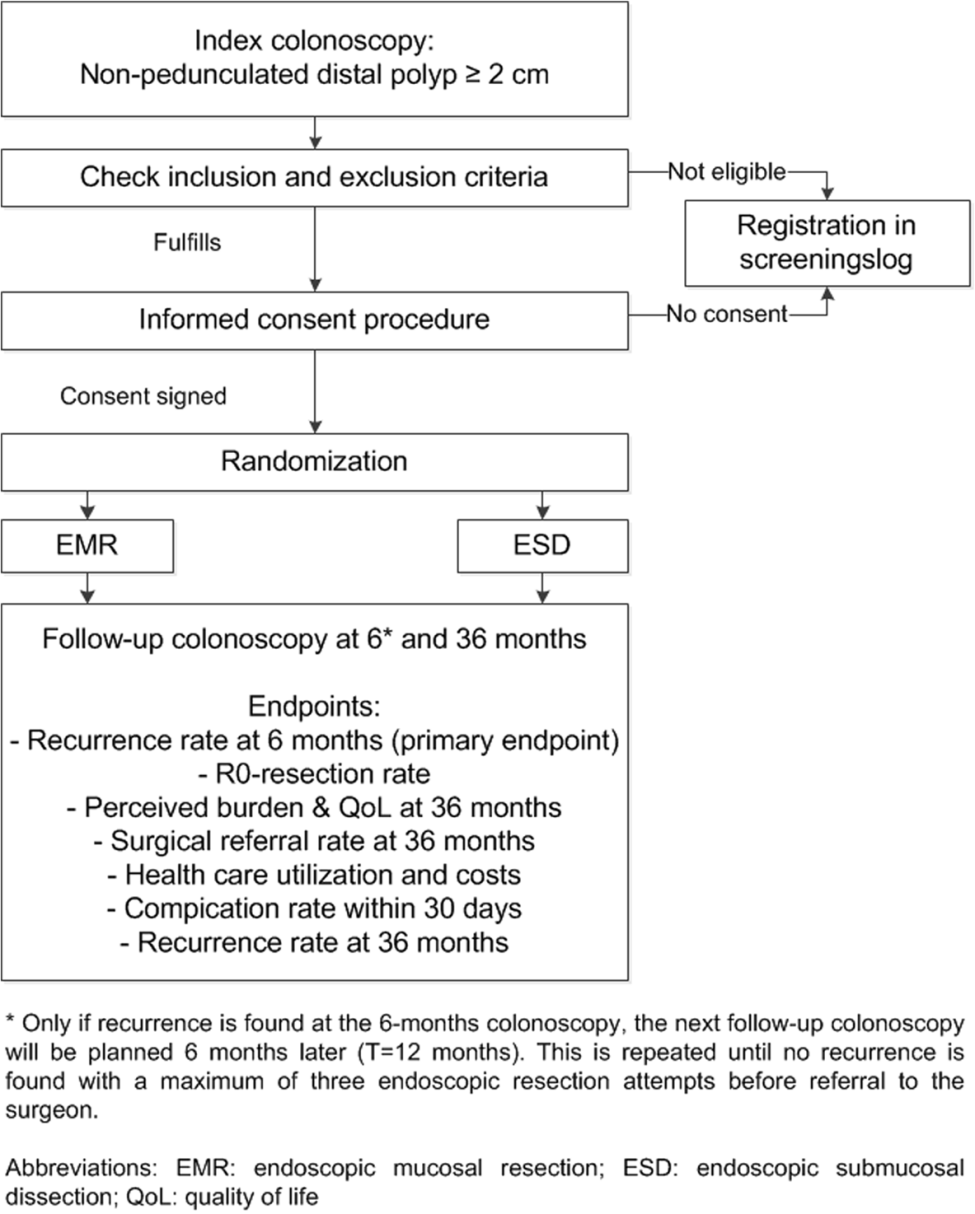
Flowchart of the study design of the MATILDA-trial

This randomized clinical trial will randomize between ESD and EMR in patients with large distal non-pedunculated colorectal adenomas. Seventeen Dutch hospitals will participate, including three academic and fourteen non-academic hospitals. The flowchart of the design of the MATILDA-trial is displayed in Fig. 1.

### Study population

The eligible study population consists of adult patients with a non-pedunculated polyp larger than 20 mm in the rectum, sigmoid, or descending colon found during screening, surveillance or diagnostic colonoscopy

Inclusion criteria:

- non-pedunculated polyp larger than 20 mm in the rectum, sigmoid or descending colon found during colonoscopy
- indication for endoscopic treatment
- ≥18 years old
- Written informed consent

Exclusion criteria:

- suspicion of malignancy, as determined by endoscopic findings (invasive Kudo pit pattern, Hiroshima type C) [12, 13] or proven malignancy at biopsy
- prior endoscopic resection attempt
- presence of synchronous distal advanced carcinoma that requires surgical resection
- the risk exceeds the benefit of endoscopic treatment, such as in patients with an extremely poor general condition or a very short life expectancy
- the inability to provide informed consent

### Study setting

The endoscopists participating in this trial are selected based on predefined criteria. With the knowledge that the operational difficulty of colon ESD is very high, a minimum prior number of procedures was defined in order to avoid that results will be biased by a learning curve. Based on the literature, 25 colorectal ESD-procedures is considered to be required to achieve expert experience [14–16]. Previous esophageal and stomach ESD experience alone will not be enough to ensure colorectal ESD expertise, as colorectal ESD is known to be technically more difficult than upper gastro-intestinal ESD due to the more challenging anatomical characteristics of the colon (thin wall, folds, peristalsis, angulated positions, and fecal fluid) [17]. This study will therefore only allow endoscopists that have performed >25 colorectal ESD procedures in the past three years to treat patients randomized to the ESD arm. In centers without ESD-experienced endoscopists, patients will be referred to an ESD-expert center when randomized to the ESD-arm. Patients randomized to the EMR arm will be treated by endoscopists which have extensive experience with EMR, defined as >500 prior EMR’s of which >50 in colorectal adenomas larger than 20 mm.

### Recruitment and randomization

Initial recruitment of patients will be performed by the local coordinating investigator of the participating center. When a polyp is found at index colonoscopy that fulfills the inclusion criteria, exclusion criteria will be checked. In presence of exclusion criteria, this will be registered in the screening log. In absence of exclusion criteria, the local coordinating investigator will provide oral and written information on the study to the patient. Patients will have as much time as they like to think about participation and will have the chance to ask any questions on the study. Thereafter the informed consent form is signed. In case of non-participation, this will be recorded in the screening log (see Fig. 1). Independent computer-based randomization will be performed (Castor Electronic Data Capture (EDC), CIWIT b.v.) in a 1:1 ratio. Randomization will be stratified by the size of the polyp (20-30 mm, 31-40 mm, >40 mm), localization (rectum, sigmoid, descending colon) and center using block sizes of five per block. Due to the nature of the treatment, neither patients nor endoscopists participating in this study will be blinded.

### Outcome measures

A schedule of the study procedures with the outcome measures is presented in Table 1.

**Table 1:**
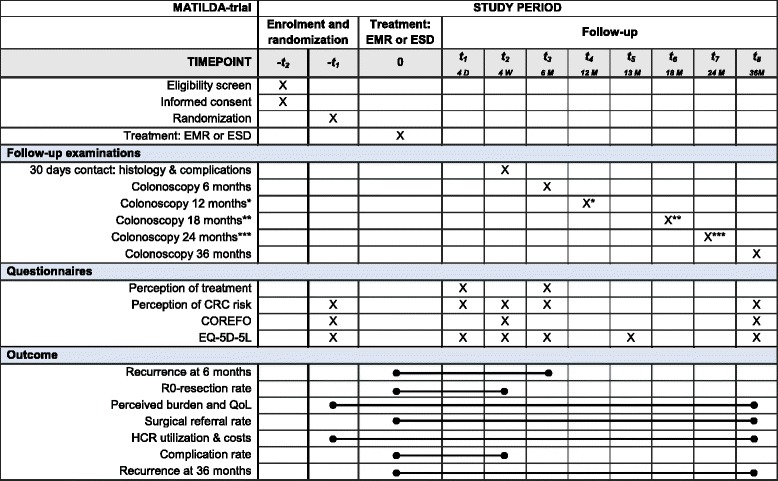
Schedule of the study procedures

*COREFO* Colorectal Function Outcome, *CRC* colorectal cancer, *D* days, *EMR* endoscopic mucosal resection, *EQ-5D-5 L* EuroQol-5 dimensions-5 levels, *ESD* endoscopic submucosal dissection, *HCR* health care resources, *QoL* quality of life, *M* months, *W* weeks; * only performed when recurrence is found at the 6 month colonoscopy; ** only be performed when recurrence is found at the 6 and 12 month colonoscopy; *** only performed when recurrence is found at the 6 and 12 and 18 month colonoscopy

#### Primary outcome

1. Recurrence rate at follow-up colonoscopy after six months. This will be observed from resected residual disease or, if not present, from biopsies of the post-polypectomy scar [18].

#### Secondary outcomes

1. Radical (R0-)resection rate. This is defined as dysplasia free vertical and lateral resection margins at histology.
2. Perceived burden and quality-of-life (QoL) among patients at 36 months. This will be assessed using questionnaires. A summary of the investigated domain and type of questions can be found in Table 2. In short, colorectal cancer anxiety is measured at baseline, 4 days and 4 weeks after ESD/EMR, and after the 6 and 36 months follow-up colonoscopy using an instrument originally designed and validated in breast cancer patients, and subsequently used to evaluate patients’ perception of colorectal cancer risk [19, 20]. Burden of the procedure will be measured on day 4 after ESD/EMR and after the 6 months follow-up colonoscopy using an instrument proven to be a reliable and valid assessment in prior studies [21–23]. Functional complaints will be measured at baseline, 4 weeks after ESD/EMR, and after the 36 months follow-up colonoscopy using the Colorectal Function Outcome (COREFO) instrument [24]. Quality-of-life will be measured at baseline, 4 days and 4 weeks after EMR/ESD, after the 6 months follow-up colonoscopy, 13 months after EMR/ESD, and after the 36 months follow-up colonoscopy using the EQ-5D-5L-instrument [25]. The EQ-5D, developed by the EuroQol group, will be used to calculate quality adjusted live years (QALYs) and consists of two parts, the EQ-5D descriptive system and the EQ visual analogue scale (EQ-VAS). The EQ-VAS records the participant’s self-reported health on a VAS from 0 (labeled “worst imaginable health state”) to 100 (labeled “best imaginable health state”). The EQ-5D descriptive system consists of five domains (i.e. mobility, self-care, usual activities, pain/discomfort and anxiety/depression) and five functioning levels (i.e. no problems, slight problems, moderate problems, severe problems or unable to function). The questionnaires will be send digital to the participating patients by Castor EDC (CIWIT b.v.). A reminder will be send when participants don’t respond. Patients who prefer to complete the forms on paper will receive the questionnaires by mail.

**Table 2:** Patients’ perceived burden and quality of life assessment

	Item	N	Method of measurement	Scale
Patients’ perception of treatment	Burden of the procedure	3	Verbal measure	5-point scale
	Burden afterwards	3	Verbal measure	5-point scale
	Overall perception	1	VAS	1-10
Patients’ perception of CRC risk	CRC Risk Perception	3	1. VAS	1. 1-100
			2. Verbal measure	2. 7-point scale
			3. Comparative measure	3. 3-point scale
	CRC Worry	2	Verbal measure	7-point scale
Colorectal Functional Outcome	Incontinence	9	Frequency measure	5-point scale
	Social impact	9	Frequency measure	5-point scale
	Frequency	2	Frequency measure	5-point scale
	Stool-related aspects	3	Frequency measure	5-point scale
	Need for medication	3	Frequency measure	5-point scale
EQ-5D-5 L instrument	Quality of life	5	Verbal measure	5-point scale
	Overall health status	1	VAS	0-100

Abbreviations: *CRC* colorectal cancer, *N* number of questions per item, *VAS* Visual Analogue Scale
3. Health resources utilizations and costs at 36 months. Data on healthcare utilization will be gathered in the electronical case record form for instruments used, time needed to perform the procedure, admission to the ward and/or intensive care, length of hospital stay, (repeated) treatment or (prolonged) hospital stay for complications, used anesthesia and anesthesia staff, material and time needed for repeated treatment or (prolonged) hospital stay for recurrence and surgical referral. These data will be used to calculate healthcare costs.
4. Surgical referral rate at 36 months. This is defined as the total number of patients that are referred for surgical management.
5. Long-term recurrence rate at follow-up colonoscopy after 36 months. This will be observed from resected residual disease or, if not present, from biopsies of the post-polypectomy scar.
6. Complication rate within 30 days after treatment. Intraprocedural perforation is defined as the condition in which the abdominal cavity is visible from the colorectal lumen during the procedure because of mural tissue defects, that requires (1) (prolonged) admission or (2) surgery. Intraprocedural bleeding is defined as bleeding that occurs during the procedure that is not controlled by electrocoagulation and/or hemoclipping, and that requires (1) transfusion or (2) termination of the endoscopic resection. Postprocedural bleeding is defined as bleeding within 30 days after the procedure resulting in (1) new presentation at the hospital, (2) hospital admission, or (3) repeated colonoscopy to obtain hemostasis. Postprocedural perforation is defined as perforation within 30 days after the procedure that is detected after completing of the procedure during which perforation did not occur, diagnosed by abdominal pain with focal guarding and a rise in C-reactive protein and/or fever (T >38.5 C) in combination with free air in the peritoneal cavity at abdominal CT-scan. Postprocedural serositis is defined as abdominal pain with focal guarding and a rise in C-reactive protein and/or fever (T >38.5 C) within 30 days after the procedure, but without signs of perforation (free air at abdominal CT-scan) and in the absence of another infection focus (urinary, pulmonary etcetera).

### Trial interventions

1. *EMR-arm*

Dose and type of sedation to be given is at the discretion of the endoscopist and will be registered. All colonoscopies will be performed with a high-resolution magnifying video-endoscope. A colloidal solution (such as succinylated gelatine) and dye will be used as the injection fluid, mixture with 1:100.000 adrenaline is optional. The purpose of this injection is to elevate the lesion away from the muscle layer, and to accentuate the plane of excision so that a wide and deep excision is achieved. Marking of the periphery of the polyp with coagulation is allowed to optimize the attempt of an en-bloc or R0-resection. A snare is then passed through the channel and opened around the lesion. The snare is snugged around the lesion and pulled. Cautery is applied to resect the lesion. Only when en-bloc resection is not feasible, the endoscopist is allowed to perform the resection in a piecemeal fashion (pEMR) in as less pieces as possible. The number of pieces will be registered. In case of pEMR, adjunct therapy with either tipping with the snare using forced coagulation (ERBE VIO 300; 25 W) or treatment with argon plasma coagulation (ERBE VIO 300; 60 W, 2.0 L/min) will be performed. This will be applied in short bursts to coagulate the entire edge of the polypectomy site. Any remaining tissue in the polypectomy site will also be coagulated. In case of en-bloc EMR, adjunct therapy with coagulation will only be performed when remnant tissue is suspected and must be registered.

2. *ESD-arm*

Dose and type of sedation to be given is at the discretion of the endoscopist and will be registered. All colonoscopies will be performed with a high-resolution magnifying video-endoscope. A 0.9 % saline solution or succinylated gelatine together with dye will be used as the injection fluid. A circumferential incision will be made using a ESD-knife. The incision must be placed on a distance of 2-5 mm around the border of the polyp. This is because thermal damage otherwise makes it difficult to evaluate the histological resection margins after resection. A complete or partial circumferential incision is performed first and then further dissection is performed after the lesion is adequately situated. The endoscopist is allowed to perform the resection using the hybrid ESD (hESD) technique only as an escape method. This hESD technique consists of a circular incision around the lesion, with partial preparation in the submucosal layer that is sufficient to capture it with a snare in a single piece. Adjunct therapy with either tipping with the snare using forced coagulation (ERBE VIO 300; 25 W) or treatment with argon plasma coagulation (ERBE VIO 300; 60 W, 2.0 L/min) will only be performed when remnant tissue in suspected and must be registered.

3. *Both arms*

For both procedures length of the procedure will be measured, defined as the total time needed for resection of the polyp, measured from the minute the injection fluid is injected until the endoscopist finishes final inspection of the resection wound and all specimen pieces are collected and removed.

For both procedures the opposite colonic wall of the resection site will be marked with India ink in case the adenoma is located in the sigmoid or descending colon, to ensure that the post-polypectomy scar can be found during follow-up.

For both procedures the following events are considered standard care, however, will be registered. In case intraprocedural perforation occurs, this will be treated using clips. In case of a minor bleeding from a small vessel, contact coagulation with the tip of a knife or coagulation with hemostatic forceps will be used for hemostasis. In cases of a severe bleeding from a large vessel or artery, hemostatic forceps will be used for hemostasis. If a pulsating large vessel is exposed within the resection wound, clipping can optionally be used to prevent delayed bleeding. If overnight admission is required, this will be registered including motivation.

### Handling of the resected specimen

The resected specimen will be pinned on a paraffin, rubber or cork sheet so that the mucous membrane surrounding the lesion is evenly flattened and the mucous membrane surface can be observed, unless fragmentation as a result of piecemeal resection hampers this. In order to prevent autolysis after resection, the specimen must be fixed as quick as possible. To prevent drying, it will be soaked in a formalin solution. Thereafter, the endoscopist is required to appropriately display the specimen so that the difference between the specimen and the clinical images is minimized and the tumor margin of the specimen can be judged. The endoscopists will provide documentation (an explanatory text) to the pathologist so that the basic information on preoperative diagnosis, the site and morphology of the lesion, and the tumor size can be accurately conveyed.

### Histopathological evaluation

The received specimen is fixed with a 4 % buffered formaldehyde solution for 24 h at room temperature. After fixation, the specimen is photographed and inked. A tangent that touches the focus closest to the horizontal tumor margin is assumed. The first cut is carried out in the direction perpendicular to the tangent. The specimen is sectioned into slices at intervals of 2 mm parallel to the first cut. All slices are embedded in cassettes for histological diagnosis. In case of long slices (>2 cm), the slice is cut in half and both halves are embedded after ink is applied at the cut edge.

Histological diagnosis of tumors is carried out in accordance with the Vienna classification of gastrointestinal neoplasia [26]. The histological type and resection tumor margins in mm (horizontal and vertical) of the lesion will be judged. Incomplete (R1) resection is defined as dysplasia infiltration of the margins and/or if infiltration cannot be determined because of coagulation artefacts, as in piecemeal resection.

### Follow-up colonoscopies

A follow-up colonoscopy is performed 6 and 36 months after the procedure for all patients. The post-polypectomy scar is checked for residual disease. In case of macroscopic residual disease this is resected and send for pathology. If not**,** three biopsies of the scar will be taken. If no recurrence is found at the 6-months colonoscopy, the next colonoscopy will be planned at 36 months. Only if recurrence is found at the 6-months colonoscopy, the next follow-up colonoscopy will be planned 6 months later (T = 12 months). This is repeated until no recurrence is found with a maximum of three endoscopic resection attempts before referral to the surgeon. Length of the follow-up colonoscopies will be registered for all time points, measured from the introduction of the scope until the endoscopist finishes final inspection of the postpolypectomy scar (including taking biopsies and/or resection of residual disease).

### Safety monitoring

The investigators will inform the subjects and the reviewing accredited medical ethics committee if anything occurs, on the basis of which it appears that the disadvantages of participation may be significantly greater than was foreseen beforehand. The study will be suspended pending further review by the accredited medical ethics committee, except insofar as suspension would jeopardize the subjects’ health. The investigators will take care that all subjects are kept informed.

Adverse events (AE) are defined according to the complication registration of the Dutch Society of Gastrointestinal Diseases (NVMDL) for non-severe complications [27]. Endoscopic resection related AE’s are: intraprocedural perforation, intraprocedural bleeding, postprocedural bleeding or postprocedural serositis that requires (prolonged) admission <10 days and/or maximum 4 units blood transfusion and/or endoscopic or percutaneous (re-) intervention. Serious adverse events (SAE) are defined as intraprocedural perforation, intraprocedural bleeding, postprocedural bleeding or postprocedural serositis or any other event with a possible or definite causal relation with the study intervention as judged by the treating physician that requires (1) > 10 days (additional) admission and/or (2) 4 units blood transfusion and/or (3) angiographic or surgical intervention and/or, (4) ICU admission and/or (5) death. These SAE’s will be reported to the accredited medical ethical committee of the University Medical Centre Utrecht (UMC Utrecht), within 15 days after the local investigator has first knowledge of the serious adverse events.

### Quality control

Datamanagement of this study will be conducted by the Netherlands Comprehensive Cancer Organisation (IKNL). Quality of data entry will be ensured through the electronical medical record, in which validation messages for users are created. In this way, a message will be shown to the data managers when something is incorrectly filled in (for example: date in the past or future, unreliable low or high patient age). Furthermore, the conduct of the MATILDA-trial will be supervised through on-site monitoring. An initiation monitor visit will be planned for all participating centers at the start of the inclusion period. Second, every center will be visited by an independent monitor after the first five included patients, and will be checked on withdrawal of informed consent, source data verification, whether inclusion and exclusion criteria are correctly followed and whether SAE’s are correctly reported. On-site monitoring later in the trial will be done if necessary. In this way, high quality will be ensured throughout the study.

### Study integrity

The medical ethical committee of the UMC Utrecht has reviewed the study in accordance with the Dutch Medical Research Involving Human Subjects Act (WMO) and other applicable European regulations and has granted a positive judgement. In addition, this study protocol has been peer reviewed by external reviewers from the Dutch Cancer Society (KWF Kankerbestrijding), the funder of this trial.

### Sample size calculation

The sample size is calculated for the primary outcome parameter recurrence rate at 6 months. Sample size for recurrence rate is calculated based on the assumption that the recurrence rate is 2 % in the ESD group and 12 % in the EMR group [8, 10, 16]. With a power of 80 % and a two-sided α of 0.05, the total number of patients needed is 198. To correct for patients lost-to-follow-up (7 %), a final total of 212 patients will be included; 106 patients in each arm.

### Data analysis

Primary analyses will be conducted on the intention-to-treat principle. Normally distributed continuous variables will be expressed as mean (± standard deviation) and not-normally distributed variables will be expressed as median (interquartile range). Categorical data will be presented with percentages. The outcome measures recurrence rate, R0-resection rate, surgical referral rate, and complication rate will be compared using a stratified analysis with a test for binary outcomes. For the perceived burden and quality of life analysis, data will be collected, presented and compared according to the user guides for the selected questionnaires [19, 24, 25, 28]. QALY estimates for the first 6-month follow-up period (i.e. short-term) and the 36-month follow-up period (i.e. long-term) will be calculated for both arms, using the Dutch tariff and the self-reported EQ-indexes at the different contact moments [29]. An area under the curve (AUC) approach will be followed by interpolating between the observations provided by the patients. Effects will be discounted at 1.5 %, according to current Dutch economic guidelines [30, 31]. QALY estimates will be compared between both arms using non-parametric test when not normally distributed. Healthcare costs will be calculated by multiplying used healthcare services by the appropriated unit cost prices. Own bottom-up micro costing will be applied for ESD, EMR and colonoscopies. For all other healthcare services reference prices will be used, where available [30, 31]. Costs will be discounted with 4 % according to current Dutch economic guidelines [30–32]. All costs will be expressed in 2018 euros. The actual healthcare costs occurring in both arms will be compared for the 6-month follow-up (i.e. short-term) and the 36-month follow-up (i.e. long-term), respectively. Costs estimates will be compared between both arms using a non-parametric test. A *p*-value of < 0.05 will be considered significant.

### Economic evaluation

In the economic evaluation, the balance between costs and effects will be assessed between both arms on both short (i.e. 6 months) and long-term (i.e. 36 months). Results of both cost and effect measurement will be integrated and expressed as incremental cost-effectiveness ratios (ICER) showing additional costs per recurrence free patient and as ICER showing incremental costs per incremental QALY. Both sensitivity and scenario analyses will be conducted. Sensitivity analysis will be conducted for uncertain parameters, and scenario analysis will be used to model for example the impact of anticipated changes of reduced frequency or prolonged interval of follow-up colonoscopies after R0-resection.

## Discussion

This will be the first randomized clinical trial comparing ESD and EMR for large distal non-pedunculated polyps. This study will not only provide information on the effectiveness and safety of both treatment strategies, but will also give insights in perceived patient burden, quality of life, the costs and cost-effectiveness of both treatments. Given that all patients in this study will be followed for a total duration of 36 months, we will be able to evaluate both short-term (i.e. 6 months) as well as long-term (i.e. 36-months) outcome measures.

We decided to include only patients with distal polyps in this trial. Not only because we expect that the risk profile for ESD will be more favorable in the rectum compared to the right colon, but also because most experience with ESD in the Netherlands currently exists with distal lesions [33]. We decided to include only non-malignant neoplasia in this trial, as we think it is unethical to randomize patients with suspected T1 CRC to the EMR arm. Given that EMR in polyps exceeding 20 mm in size is most often performed in a piecemeal fashion, inclusion of patients with suspected T1 CRCs in this trial would undoubtedly result in unnecessary referral of low risk T1 CRCs for surgery due to positive resection margins [34].

With regard to the outcome measures, we chose to evaluate the primary outcome measure recurrence rate after a duration of 6 months. From literature, it is known that about 76 % of recurrences after EMR occur after 3 months, increasing to 96 % after 6 months [35]. Thus, 6 months seems the moment we are able to catch the vast majority of the recurrences. However, studies that looked at long-term recurrence rates also found that recurrence can occur up to 36 months after EMR [35–37]. Therefore, we feel a second outcome measure for long-term follow-up is obligatory for this trial. R0-resection was chosen as secondary outcome measure as this might be an important factor to decide for the frequency and interval of follow-up colonoscopies in future patients. If our assumption is correct that a R0-resection does not show recurrence at long-term follow-up, this will offer an argument to prolong the interval and frequency of follow-up colonoscopies after ESD with R0-resection. Last, we decided only to apply a healthcare payer perspective when conducting the cost-effectiveness analysis. Argument is that most of the patients will be above 65 years and thus retired, absence from work and/or lower efficiency while at work (i.e. productivity losses) will be rare. Adding an additional questionnaire for investing potential work losses from unpaid work were considered too burdensome, and not pay off for the increased risk of early termination.

In the development of the study design, sources of bias were attempted to be minimized. Stratified randomization will be performed in order to prevent confounding. Moreover, in order to pre-empt confounding due to differences in operator experience between the groups, we decided only to allow endoscopists with sufficient prior experience to participate in this trial. Threshold values for participation were determined based on previous published literature and are defined in the study protocol [14–16]. Due to the nature of the treatment, neither patients nor endoscopists participating in this study will be blinded. In order to avoid information bias in the endoscopists’ evaluation of recurrence, the study protocol mandates endoscopists to take biopsies of the post-polypectomy scar. In this way, an objective evaluation is guaranteed.

This study will support an optimal use of health resources in the future and will have direct implications for future patients with large colorectal polyps. Colorectal cancer screening programs are expected to further increase the detection rate of large colorectal lesions, making it even more important to put effort in research on the optimal resection technique.

## Abbreviations

AE, adverse event; Castor EDC, Castor electronic data capture; CCMO, Central Committee on Research Involving Human Subjects; COREFO, Colorectal Function Outcome; CRC, colorectal cancer; EMR, endoscopic mucosal resection; EQ-5D-5 L, EuroQol-5 dimensions-5 levels; ESD, endoscopic submucosal dissection; hESD, hybrid endoscopic submucosal dissection; ICH-GCP, good clinical practice guidelines; IKNL, Netherlands Comprehensive Cancer Organisation*;* NVMDL, Dutch Society of Gastrointestinal Diseases; pEMR, piecemeal endoscopic submucosal resection; QALY, quality adjusted life year; QoL, quality of life; SAE, serious adverse event; UMC, University Medical Centre Utrecht; VAS, visual analogue scale; WMO, Dutch Medical Research Involving Human Subjects Act
